# Improved Detection of Rare HIV-1 Variants using 454 Pyrosequencing

**DOI:** 10.1371/journal.pone.0076502

**Published:** 2013-10-02

**Authors:** Brendan B. Larsen, Lennie Chen, Brandon S. Maust, Moon Kim, Hong Zhao, Wenjie Deng, Dylan Westfall, Ingrid Beck, Lisa M. Frenkel, James I. Mullins

**Affiliations:** 1 Department of Microbiology, University of Washington, Seattle, Washington, United States of America; 2 Children’s Hospital Research Institute, Seattle, Washington, United States of America; 3 Department of Pediatrics, University of Washington, Seattle, Washington, United States of America; 4 Department of Laboratory Medicine, University of Washington, Seattle, Washington, United States of America; 5 Department of Medicine, University of Washington, Seattle, Washington, United States of America; British Columbia Centre for Excellence in HIV/AIDS, Canada

## Abstract

454 pyrosequencing, a massively parallel sequencing (MPS) technology, is often used to study HIV genetic variation. However, the substantial mismatch error rate of the PCR required to prepare HIV-containing samples for pyrosequencing has limited the detection of rare variants within viral populations to those present above ~1%. To improve detection of rare variants, we varied PCR enzymes and conditions to identify those that combined high sensitivity with a low error rate. Substitution errors were found to vary up to 3-fold between the different enzymes tested. The sensitivity of each enzyme, which impacts the number of templates amplified for pyrosequencing, was shown to vary, although not consistently across genes and different samples. We also describe an amplicon-based method to improve the consistency of read coverage over stretches of the HIV-1 genome. Twenty-two primers were designed to amplify 11 overlapping amplicons in the HIV-1 clade B *gag-pol* and *env* gp120 coding regions to encompass 4.7 kb of the viral genome per sample at sensitivities as low as 0.01-0.2%.

## Introduction

Pyrosequencing using the Roche/454 platform has been used to study HIV-1 transmission [1,2], emergence of drug resistance [3-5], and superinfection [6,7]. Pyrosequencing allows many relatively long DNA templates to be sequenced in parallel (~350nt with product literature promising >600bp), generating more than 1 million sequences in a single run. However, the promise of “deep” sequencing of variable sequence populations such as within HIV infections, using MPS on the 454 and other platforms, has been slow to materialize in part because of the complexities introduced by PCR. Nested PCR, often involving 70 or more rounds of amplification of the typically low abundance HIV target molecules, is often required to obtain sufficient numbers for detection and subsequent use in these instruments. Substitution errors that occur during these initial PCR steps will be observed following parallel sequencing of the target molecules, and, in most cases, cannot be reliably distinguished from true viral genetic variation.

Accurate detection and quantitation of low-frequency variants is dependent on four main factors: quantitation of amplifiable input template molecules, removal of artifactual errors that occur during the pyrosequencing reactions (homopolymer length variation and carry-forward errors), minimization of misincorporation error rates during preliminary PCR steps, and consistent and adequate read coverage of the sample population.

In the case of HIV infection, plasma viral loads are determined using clinical assays using standard controls to account for inefficiencies in the extraction and reverse transcription of nucleic acids and compounds in the specimen that inhibit amplification. Thus, reported values are often extrapolations of the actual number of genome fragments amplified [8,9]. Furthermore, the highly conserved regions amplified by commercial assays are seldom of interest in research studies. Following clinical viral load assay a sample might also experience multiple freeze-thaw cycles that will degrade virions and viral RNA. PCR efficiency is also highly dependent upon the specificity of the primers, the PCR conditions, and the length of the amplicon. Hence, in calculating the number of amplifiable templates, it is crucial to use the exact PCR conditions that will be employed for acquiring sequencing templates. Without careful estimation of the number of amplifiable input molecules, an incorrect representation of the population diversity will result [10-12].

Due to miscalled lengths of homopolymer runs, pyrosequencing results in a very large number of errors by creating artifactual insertions or deletions (InDels) in the sequence reads. Several algorithms have been put forth to remove most of these errors [13-17]. To allow real variants to be distinguished from errors that occurred prior to pyrosequencing, a frequency threshold above the error rate of PCR should be defined. Errors resulting from both PCR and pyrosequencing have been well described [18-20]. Misincorporation errors during pyrosequencing are typically not a problem, as thousands of templates are extended on individual beads during each flow cycle, and most will incorporate the correct base. The mean mismatch error rate of 454 without any PCR has been reported to be 0.02% which is significantly lower than the error-rate when PCR is used to amplify samples prior to 454 sequencing [19]. Therefore, the biggest source of mismatch error in 454 sequencing comes from the PCR used to initially amplify the samples.

Thus, pyrosequencing errors primarily result in miscalls of the length of homopolymers, while PCR amplification of templates prior to 454 is associated with substitution errors. Distinguishing background PCR misincorporation errors from real low-level variation represents a significant obstacle for the analysis of pyrosequencing data. Some publications have noted the problem of miscalling PCR and pyrosequencing errors as real variants [21,22], and a variety of computational methods have been developed that attempt to distinguish real variation from error [13-17]. Some of these computational methods rely on flowgram or quality score information to infer whether a variant is real. Since quality scores and flowgrams are limited to the probability of only errors that occur during pyrosequencing, they cannot be used to correct substitution errors that accumulate during the PCR stage before emPCR. Application of random bar codes on cDNA or PCR primers allows for determination of a consensus for each sequence, and thus will obviate much of the error that occurs during the initial PCR steps. However, this approach is only practical for short amplicons that include the 3’ end or both ends of the template [23,24].

Lastly, to detect low-frequency variants, there must be sufficient sequence coverage. The term “coverage” has sometimes been used to mean the number of reads obtained in the sequencing run. However, the number of reads cannot be taken to indicate the number of templates sequenced, unless there are fewer reads than the initial number of amplifiable templates in the PCR reaction and unless each read encompasses the full length of the amplicon being sequenced. Even when the number of reads is fewer than the number of templates, each read can only approximate the actual template sequence due to PCR and pyrosequencing errors. Our approach involves over-sequencing each template, and then using a frequency cutoff, derived from the number of templates, to estimate the detection limit of rare variants. Therefore, our usage of “coverage” indicates the degree of over-sequencing of each template, and is defined as the number of reads at a particular position divided by the number of templates.

Coverage by either definition varies by position in a template, because the DNA is often sheared to build libraries for pyrosequencing. Shearing techniques tend to produce uneven read coverage, in which one position might have thousands of reads, while another position a short distance away might have only a few reads. A comparison of three different library shearing methods found regions of uneven read coverage was an issue in all three [25].

The methods employed here included an attempt to overcome the problem of uneven coverage by creating a series of overlapping amplicons of approximately the length of pyrosequencing reads. We designed a total of twenty-two 2^nd^ round primers to amplify 11 overlapping amplicons that span the *gag-pol* and *env* gp120 coding sequences of HIV-1. While such methods could be applied to any template population or any region of HIV-1, we focused on developing a method for sequencing these regions because of their use as immunogens (gag) and control regions (env) in a recent HIV vaccine clinical trial [26,27].

We report methods for increasing the sensitivity of detection of low-level variants in sequence populations through optimization of PCR conditions and choice of DNA polymerases. Through these improvements we have reduced the threshold of detection of low-level variants to 0.02-0.1%.

## Methods

### RNA extraction and cDNA synthesis

HIV-1 RNA was extracted from the plasma of infected individuals using the QIAamp Viral RNA Mini Kit (Qiagen, Valencia, CA) according to the manufacturer’s protocol. A total of 560µL plasma was extracted and eluted in 80µL elution buffer. cDNA was synthesized using 10 µl of RNA and the Takara BluePrint First Strand Synthesis Kit (Clontech 6115A) according to the manufacturer’s protocol. cDNA was synthesized with gene specific primers, R3337-1 (5’- TTTCCYACTAAYTTYTGTATRTCATTGAC-3’) for *gag-pol* and R9048 (5’-AGCTSCCTTGTAAGTCATTGGTCTTARA-3’) for *gp120*, at 400nM final concentration.

### PCR and sequencing

First-round reactions used 2,000 copies of pNL4-3 [28], a plasmid containing a full-length HIV-1 genome, as the template, except in the case of the enzyme sensitivity comparison in which we used a patient plasma sample as a source of viral RNA. We also applied our methods to specimens from five additional subjects recruited through the University of Washington HIV Primary Infection Clinic [29]. The plasma viral loads for these 6 specimens are shown in Table S1. To sequence a 2.6 kb fragment of the HIV-1 *gag* -*pol* gene region and a 2.1 kb fragment of *env*, first- and second-round PCRs were performed using Kapa HiFi HS (Kapa Biosystems, Boston) (Table 1). In brief, first-round PCR reactions were conducted in two separate 25 µL reaction volumes with 1-6 µL of cDNA as template, corresponding to 200 to 500 input template copies based on limiting endpoint dilution PCR using the template estimator program Quality [10] (http://indra.mullins.microbiol.washington.edu/quality/). Second-round PCR reactions were conducted in 25 µl, using 2 µl of the first round reaction as template. A total of five multiplex PCR and one *Monoplex* PCR were performed to generate 11 overlapping PCR amplicons ranging from 384-603 bp in size. Primer sets and sequences are listed in Table 2. To accommodate the exceptional sequence diversity of HIV genomes, we utilized nucleotide frequencies across the HIV genome derived from the Los Álamos HIV database to design our primers to bind to conserved regions of the HIV genome. Second-round PCR reaction products were pooled and purified using AMPure beads according to the manufacturer’s protocol (Agencourt, Beverly, MA). Briefly, 180 µL of PCR product was mixed with 144 µL of AMPure beads. The solution was then vortexed, incubated, washed, and purified. Finally the beads were resuspended in 60 µL of 10mM Tris-Cl. A Nanodrop instrument (Thermo Scientific; Waltham, MA) was used to determine DNA concentration and purity. The purified product was then diluted to 100ng/µL with 10mM Tris-Cl. All purified products were stored at -20°C prior to sequencing.

**Table 1:** PCR conditions.

**1^st^ Round PCR** (25 µl reaction volumes)		
**Enzyme**	**Reaction composition**	**Cycling conditions**
Advantage2	1X Adv2 buffer, 200 µM dNTP, 400 nM primers, 1X Adv2 Pol Mix	94°C 3 min. 35 cycles: 94°C 20 sec, 64°C 20 sec, 68°C 2 min. 1 cycle: 68°C 7 min. 4°C hold
Phusion	1X Master Mix, 500 nM primers	98°C 1 min. 35 cycles: 98°C 10 sec, 64°C 30 sec, 72°C 1 min. 1 cycle: 72°C 7 min. 4°C hold
KOD HS	KOD 1X buffer, 1.5 mM MgSO4, 200 µM dNTP, 400 nM primers, 0.5U KOD HS DNA Polymerase	95°C 2 min. 35 cycles: 95°C 20 sec, 64°C 20 sec, 70°C 1 min. 1 cycle: 70°C 7 min. 4°C hold
Kapa HiFi Hot Start	1X Kapa HiFi Fidelity Buffer, 300 µM each dNTP, 500 nM primers, 0.5 U Kapa HiFi HS DNA Pol	95°C 2 min. 35 cycles: 98°C 20 sec, 64°C 30 sec, 72°C 1.5 min. 1 cycle: 72°C 5 min. 4°C hold
**2^nd^ Round PCR** (25 µl reaction volumes)		
**Enzyme**	**Reaction composition**	**Cycling conditions**
Advantage2	1X Advantage 2 PCR Buffer, 280 µM dNTP, 400 nM primers, 1X Advantage 2 Polymerase Mix, 2 µl 1st round PCR reaction	94°C 3 min. 5 cycles: 94°C 15 sec, 60°C 25 sec, 68°C 10 sec. 25 cycles: 94°C 15 sec, 64°C 15 sec, 68°C 10 sec. 1 cycle: 68°C 7 min. 4°C hold
Kapa HiFi Hot Start	1X Kapa HiFi Fidelity Buffer, 300 µM dNTP, 400 nM primers, 0.5 U Kapa HiFi HS DNA Polymerise, 2 µl 1st round PCR reaction	95°C 2 min. 5 cycles: 98°C 20 sec, 58° 30 sec, 72°C 10 sec. 30 cycles: 98°C 20 sec, 64°C 30 sec, 72°C 10 sec. 1 cycle: 72°C 5 min. 4°C hold

**Table 2:** List of primers used to amplify viral DNA prior to pyrosequencing.

**1^st^ Round PCR**							
**Primer**	**Amplicon**		**Sequence**	**Amplicon Size**			
F683	gag-pol		CTCTCGACGCAGGACTCGGCTTG	2654			
R3337-1	gag-pol		TTTCCYACTAAYTTYTGTATRTCATTGAC				
F5957-1	gp120		TTAGGCATYTYCTATGGCAGGAAGAA	2096			
R8053-1	gp120		CAAGGCACAKYAGTGG**T**GCARATGA				
**2^nd^ Round PCR**							
**Primer**	**Amplicon**	**Primer Set**	**Sequence**	**Amplicon Size**	**Average # forward reads**		**Average # reverse reads**
F762	gagp17	1	TTGACTAGCGGAGGCTAGAAGGAGA	504	4861		4560
R1196	gagp17	1	TGCACTATAGGGTAATTTTGGCTGAC				
F1099	gagp24a	2	ATAGAGGAAGAGCAAAACAAAAGTAAGA	603	5173		5454
R1632	gagp24a	2	GCTRGYAGGGCTATACATYCTTACTAT				
F1550alt1	gagp24b	3	CACCTATCCCAGTAGGAGAMATYTATA	505	7954		7874
R1985	gagp24b	3	CCTTCYTTGCCACARTTGAAACAYTT				
F2195	pol1	4	AACARCARCYCCCYCTCAGAARCAGG	496	8997		10222
R2621	pol1	4	CCAYTGTTTAACYTTTGGKCCATCCATT				
F2548	pol2	1	TTCCCATTAGTCCTATTGAAACTGTAC	562	8101		7618
R3040	pol2	1	ATRCTRCWTTGGAATATTGCTGGTGAT				
F2966	pol3	5	ACCAGGGATTAGATATCAGTACAATGT	384	11116		9515
R3280	pol3	5	ATAGGCTGTACTGTCCATTTATCAGG				
F6191	env1	5	GATAGAMTAAKAGAAAGAGCAGAAGACA	502	7859		9016
R6623	env1	5	ATCMGTGCAAKTTAAAGTAACACAGAGT				
F6547alt1	env2	2	TAATCAGTTTATGGGATSAAAGYYTAAA	495	11814		12123
R6972	env2	2	CATGTGTACATTGTACTGTGCTGACAT				
F6853alt1	env3	4	TTGARCCAATTCCYATACATTAYTGTR	391	8814		9441
R7174	env3	4	AATGCTCTYCCTGGTCCYATATGTAT				
F7111alt1	env4	6	GTACAAGACCCAACAACAATACAAGRA	542	12058		13109
R7583alt2	env4	6	TADTAGCCCTGTAATATTTGATRARCA				
F7504alt1	env5	3	GGCARGARGTAGGAARAGCAATRTATG	597	7703		8605
R8031	env5	3	TGAGYTTTCCAGAGCARCCCCAAAT				

### Comparison of DNA polymerases

To compare the error rate and sensitivity of the PCR enzymes, four different DNA polymerases were used to amplify two amplicons (named gagp24b, 505bp, and env5, 597bp) within the HIV-1 genome. The enzymes used were Advantage 2 (Adv2; Clontech, Mountain View, CA), Phusion High Fidelity DNA polymerase (New England Biolabs, Ipswich, MA), KOD Hot Start DNA polymerase (EMD Millipore, Billerica, MA) and Kapa HiFi Hot Start (Kapa Biosystems; Boston, MA). First-round PCR reactions were conducted in two separate 25 µL reactions with primers (*gag-pol*: F683 5’- CTCTCGACGCAGGACTCGGCTTG-3’ and R3337-1 5’- TTTCCYACTAAYTTYTGTATRTCATTGAC-3’) and (*env* gp120: F5957-1 5’- TTAGGCATYTYCTATGGCAGGAAGAA-3’ and R8053-1 5’- CAAGGCACAKYAGTGGTGCARATGA-3’). Equal volumes of each first-round PCR were then combined, and 2 µL was used as template in 25 µL total reaction volumes in a second-round nested PCR. Multiplex second-round PCRs used one pair of primers (Primer set 3, Table 2), which targeted genes of interest within the first-round PCR amplicons. The second-round primers also contained universal adaptors A and B, as well as one of seven different MIDs described by Roche: ACGAGTGCGT, ACGCTCGACA, AGACGCACTC, AGCACTGTAG, ATCAGACACG, ATATCGCGAG, and CGTGTCTCTA (Roche, Basel, Switzerland). PCR conditions and cycling parameters for each DNA polymerase are listed in Table 1.

Second-round PCR products were visualized on a QIAxcel (Qiagen, Hilden, Germany). Confirmed positive reactions were pooled and purified using AMPure beads as outlined above.

### 454: Pyrosequencing

Briefly, each pooled and purified PCR product was quantified using the Quan-it PicoGreen dsDNA assay (Invitrogen, Carlsbad, CA). Each product was then diluted to a working stock of 10^7^ molecules/µl in TE buffer. All 11 amplicons were then pooled together in equimolar ratios into a single tube for pyrosequencing.

Emulsion PCR and sequencing were performed according to the manufacturer’s GS FLX Titanium protocols (Roche, Basel, Switzerland). PCR products were added to the emulsion PCR at a ratio of 1-2 molecules per bead. The picotiter sequencing plate was prepared with a single gasket, creating two regions. Four million enriched beads were loaded equally across the two regions. All error rate comparison data were derived from the same sequencing plate, while the patient samples were run on separate plates.

### Error Rate Calculations

First, raw sequences were screened to remove any reads that were less than 100 bp or contained the ambiguous base ‘N’. Sequences generated for each sample were then aligned to the pNL4-3 reference or a patient specific consensus using BLAST [30] with the following parameters: match: 1; mismatch: -1; gap existence: 1; gap extension: 2. We performed pairwise alignment by applying the NCBI BLASTN program to measure different types of errors (insertions, deletions and substitutions). Second, a perl script was used to parse the BLAST pairwise alignment output XML file (parseBlastXML_calcErrRate.pl, available at http://indra.mullins.microbiol.washington.edu/). For each pairwise alignment between read and the reference, the script counts the number of aligned nucleotides in the reference sequence, as well as the numbers of insertions, deletions and substitutions in the read compared to the reference. By processing all the reads that aligned to the reference, the total numbers of aligned nucleotides in the reference sequence and different types of errors were calculated. All site specific errors were then tabulated and the mean frequencies and 95% confidence intervals were calculated

### Statistical Analyses

The program Quality [10] was used to calculate the number of amplifiable templates in each sample with data generated from nested PCR following endpoint dilution (positive or negative amplifications monitored by gel electrophoresis). Quality is a web tool (http://indra.mullins.microbiol.washington.edu/quality/) based on the minimum chi method developed by Taswell [31] for limiting dilution assays.

The difference in error rates between enzymes was compared using a Kruskal-Wallis ANOVA test with Dunn’s multiple comparison correction to compare multiple enzymes simultaneously. P-values < 0.05 were considered significant. All statistical tests were conducted using GraphPad Prism 6.0 for Mac (GraphPad, San Diego).

## Results

### Sensitivity of PCR as a function of the Polymerases Used

We first compared the sensitivity of 6 different enzyme combinations by limiting dilution endpoint PCR for PIC subject 64236 (RNA) and the plasmid clone pNL4-3 (DNA) (Table 3). The sensitivity of different enzymes did vary, but not consistently across different genes or samples. A seventh combination that employed the use of Phusion in both rounds of PCR resulted in consistently low sensitivity, and was not tested further (data not shown).

**Table 3:** Sensitivity of different enzyme combinations for two different samples and genes.

**Enzyme**	**Sample**	Pol Mean c/µL (SD*)	Env Mean c/µL (SD*)
Adv2/Adv2	64236 Plasma	2.0 + 0.5	7.5 + 2.4
Phusion/Adv2	64236 Plasma	8.3 + 3.4	4.7 + 1.5
Phusion/Adv2 (25 cycles)	64236 Plasma	8.3 + 3.4	4.7 + 1.5
KOD/Adv2	64236 Plasma	2.1 + 0.5	6.0 + 2.1
Phusion/Kapa HiFi	64236 Plasma	8.3 + 3.4	4.7 + 1.5
Kapa HiFi/Kapa HiFi	64236 Plasma	6.1 + 2.1	4.5 + 1.7
Adv2/Adv2	pNL4-3	228 + 142	91 + 57
Phusion/Adv2	pNL4-3	228 + 142	228 + 142
Phusion/Adv2 (25 cycles)	pNL4-3	228 + 142	228 + 142
KOD/Adv2	pNL4-3	91 + 57	448 + 161
Phusion/Kapa HiFi	pNL4-3	228 + 142	366 + 231
Kapa HiFi/Kapa HiFi	pNL4-3	537 + 350	437 + 169

Values are in predicted number of copies of virus per microliter of DNA derived from endpoint dilution PCR. * SD = standard deviation.

### Substitution errors varied up to 3-fold with different DNA polymerases

**Figure 1:**
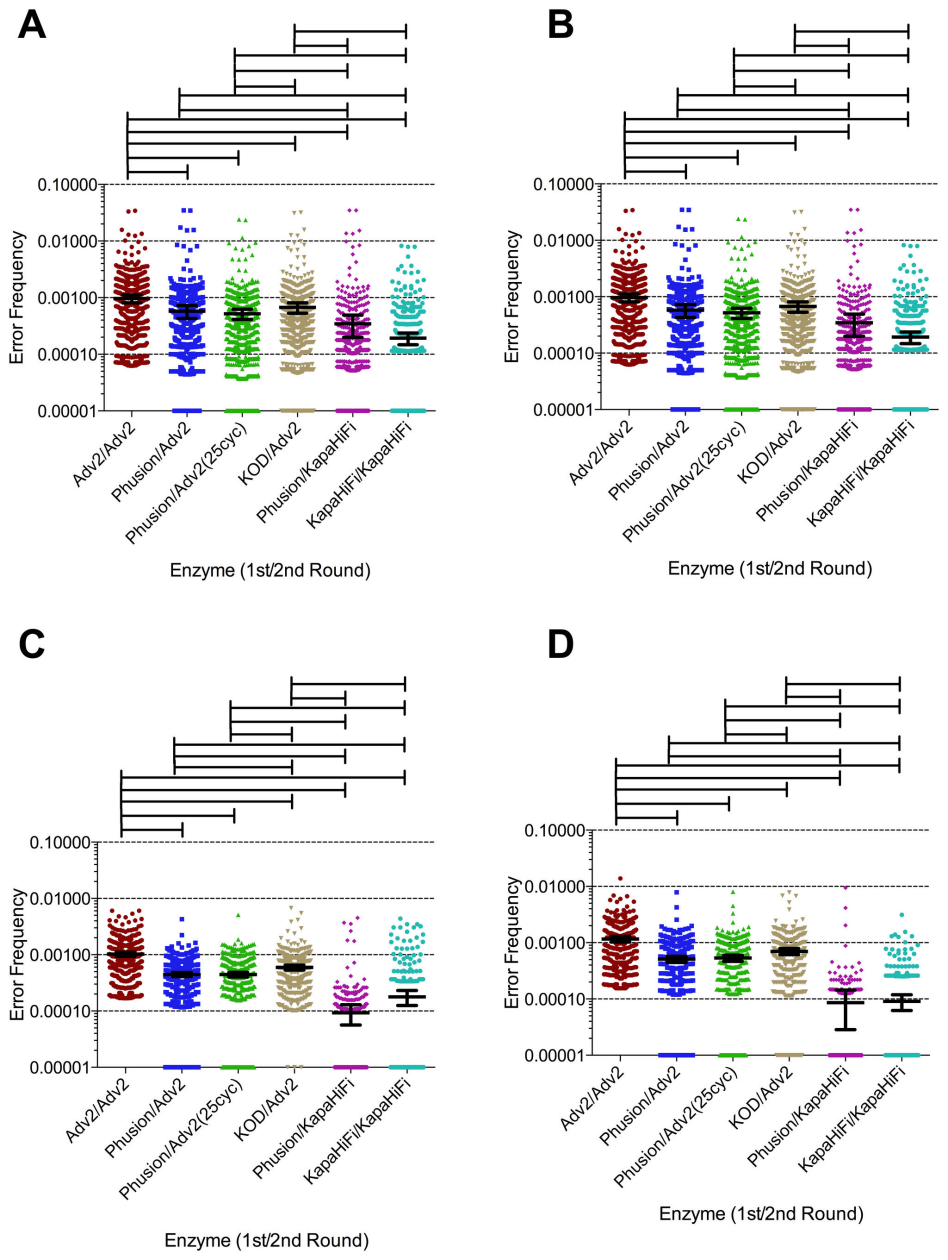
Frequency of substitution errors for DNA polymerase combinations. Raw data from a (A) 597bp amplicon from env and a (B) 505bp amplicon from gag. Reads were aligned to pNL4-3 and the frequency of all errors for each site are shown. The error frequency for each site was taken as the number of incorrect bases divided by the total number of reads. Each dot represents a substitution relative to the pNL4-3 consensus. Error corrected data from (C) env and (D) gag. Carry-forward errors were corrected using an in-house perl script as described in the Methods. Bars above each panel indicate which pairwise comparisons are significant at p<0.05. Pairwise comparisons were done by a Kruskal-Wallis test, with a Dunns test correction for multiple comparisons.

The rate of PCR error contributes to determining the sensitivity of variant detection in pyrosequencing by establishing the minimum frequency at which a true viral variant can be reliably distinguished from a PCR artifact. We therefore investigated the error rate in PCR amplification using a variety of commercially available error-correcting polymerase mixtures (Figure 1). Two different regions were amplified in the same multiplex PCR, “gagp24b” (corresponding to HXB2 positions 1576-1960) and “env5” (HXB2 7530-7974), from primer set 3. Using the small starting amounts of HIV cDNA typically available from biological samples we first attempted one round of PCR (up to 60 cycles) to amplify HIV cDNA but were unable to visualize the product on an ethidium bromide gel for the template input tested (visualization as required to calculate the number of amplifiable templates). We therefore used nested PCR to amplify sufficient levels of product.

Use of a high-fidelity enzyme in the first round of PCR lowered the raw error rate significantly for both amplicons (Fig. 1A,B). Using Advantage 2 in either the first round or both rounds of PCR (Adv2/Adv2), the error rates were 9.6x10^-4^ for gag24b and 7.4x10^-4^ for env 5. When Phusion was used in the first round (Phusion/Adv2) the mean error rate was lowered significantly, to 5.8x10^-4^ and 4.3x10^-4^, respectively; both p<0.05. However, using Phusion in the first round and Kapa HiFi HS in the second round lowered the mean error rate even further to 3.4x10^-4^ and 2.5x10^-4^, respectively; both p<0.05. This represents a 2.8-fold reduction for *gag* and a 2.9-fold reduction for *env* in the mean substitution error compared to Adv2/Adv2. Lowering the number of second-round PCR cycles from 35 to 25 had no effect on the error rate for Phusion/Adv2. Furthermore, for all enzymes tested there was no significant difference in the mean substitution error rates between the gagp24b and env5 amplicons.

We also processed the raw read data using an error correction pipeline that corrects for insertion/deletion (indel) and carry-forward errors [32]. After removing these errors, the substitution error for the corrected reads varied depending on the enzyme used (Figure 1C,D). The mean error rate after correction for Adv2/Adv2 was 5.4x10^-4^ for gagp24b and 4x10^-4^ for env5. For Phusion/KapaHiFi the mean error rate after correction was 3.6x10^-5^ and 3.4x10^-5^, respectively. When considering the upper 95^th^ percentile of the error rate Adv2/Adv2 had a rate of 2.39 x10^-3^ and 1.9 x10^-3^, respectively, while the high-fidelity enzymes Phusion/Kapa HiFi had an upper 95^th^ percentile error rate of 1.5x10^-4^ and 1.6x10^-4^, respectively (Table 4).

**Table 4:** Mean and upper 95% percentile error cut-off for different enzyme combinations.

	**Mean gag (95^th^ %ile)**	**Mean env (95^th^ %ile)**	**Mean gag corrected (95^th^ %ile)**	**Mean env corrected (95^th^ %ile)**
Adv2/Adv2	0.10 (0.31)	0.07 (0.28)	0.05 (0.24)	0.04 (0.18)
Phusion/Adv2	0.06 (0.15)	0.04 (0.12)	0.02 (0.11)	0.02 (0.09)
Phusion/Adv2 (25cycles)	0.05 (0.14)	0.04 (0.12)	0.03 (0.12)	0.02 (0.09)
KOD HS/Adv2	0.07 (0.19)	0.05 (0.13)	0.03 (0.15)	0.02 (0.11)
Phusion/KapaHiFi	0.03 (0.09)	0.03 (0.06)	0.004 (0.01)	0.003 (0.02)
KapaHiFi/KapaHiFi	0.02 (0.07)	0.02 (0.11)	0.004 (0.03)	0.007 (0.03)

Numbers shown are in %. Upper 95^th^ percentile cut-offs were chosen for determining where variants can reliably be called using different PCR enzymes.

### Read coverage using an overlapping amplicon PCR protocol

**Figure 2:**
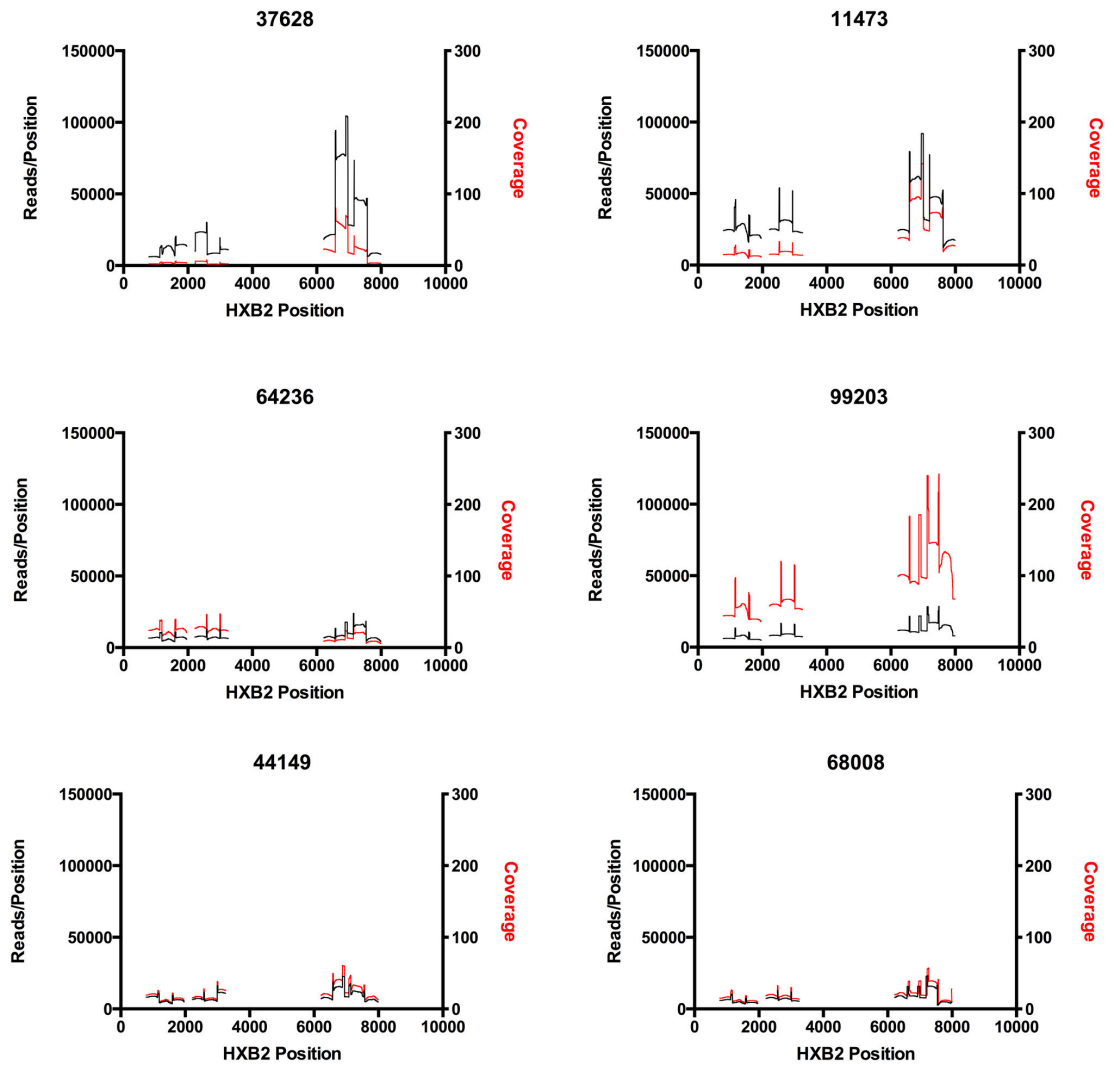
Read coverage following sequencing of amplicons derived from three regions of the viral genome from six HIV-1 infected subjects. Eleven amplicons, representing ~4.7kb of of the viral genome were aligned to a patient specific consensus. The position of each amplicons is shown relative to the HXB2 reference genome. The “spikes” in read coverage correspond to regions in which adjacent amplicons overlap. We did not have an amplicon that spanned the region around position 2000.

We used an overlapping amplicon PCR protocol to attempt to improve read coverage, which can be inconsistent after DNA shearing. First-round PCR products were amplified separately using primers that targeted a 2.6kb *gag-pol* amplicon and a 2.1kb *env* gp120 amplicon. These first-round products were then pooled and subjected to a nested second-round of PCR, resulting in 11 different amplicons, six in *gag-pol* and five in *env* gp120, ranging from 391 bp to 603 bp in size. In total, we recovered sequences across 4.7 kb of the HIV-1 genome. Examples of read coverage for the 6 PIC subjects are shown in Figure 2. Regions with apparent spikes in coverage result from the overlap of amplicons generated by pooling the alignments. As expected, the two longest amplicons, gagp24a and env5 had a mean of 21.8% and 18.4% full-length reads for the seven different subjects samples tested while the shortest amplicon, pol3 had an average of 90% of reads covering the entire amplicon. To obtain full bi-directional coverage then, shorter amplicons that match with the average read length of 454 sequencing are required.

We sought to over-sequence each template in order to ensure that the observed variation was not due to errors that occurred during pyrosequencing, targeting 10 reads per template in both directions. We generated an average of 836,817 reads for each of the four plates from an average of 40,730 input molecules, for an average of 21.5 reads per template, or 10.7 reads per template in each direction.

## Discussion

Massively parallel sequencing (MPS) technologies are being used to study HIV sequence variation and evolution. However, the ability to accurately detect low-frequency variants is dependent on quantitation of DNA input, low error rates in PCR, adequate read coverage, and correction of systematic errors, such as homopolymer length variation and carry-forward errors. Substantial effort has been devoted to computational methods to detect low-frequency variants. However, little has been done to lower the threshold of detection in the sample preparation steps. We sought to improve these protocols in order to identify real variants at the lowest possible frequencies.

While we compared the sensitivities of different enzymes combinations, PCR sensitivity appeared to be sample as well as enzyme dependent. Only the use of Phusion in both rounds of PCR showed a clearly lower efficiency. Additionally, our results demonstrated the shortcoming of relying on clinical viral load measurements to estimate the number of HIV templates that are being sequenced. In the patient samples evaluated here, the estimated number of copies was on average 4.7 times lower than the clinical viral load estimate (Table S1). This is attributable to the amplification of longer products (2.6 and 2.1 kb) for sequencing than the smaller fragments for clinical viral load estimates (<150bp) [8], the use of different primers and enzymes, and the possibility that the RNA was exposed to multiple freeze-thaw cycles since viral load measurements were done. Furthermore, clinical viral load assays correct for inefficiencies in extraction, reverse transcription, and PCR. The number of amplifiable templates in the PCR prior to 454 sequencing has a direct impact on detection of minor variants. Thus, clinical viral load is not a suitable measure to accurately estimate the number of amplifiable templates that are input into the PCR. Instead, it is necessary to specifically quantify the number of amplifiable templates using the same protocol, primers, amplicon length, and reaction conditions used to amplify the DNA template for MPS.

Next, we compared the error rates of different polymerases. It has previously been shown that the average mismatch error rate varies depending on enzyme used [18]. Interestingly, while there was a significant decrease in the mean mismatch error when a high fidelity DNA polymerase was used in the first round of PCR (e.g., Adv2/Adv2 vs. Phusion/Adv2), the error rate was lowered even further when a high-fidelity enzyme was used in both rounds (the best combination we tested being Phusion/KapaHiFi). This suggests that mismatch errors that occur in the 2^nd^ round of PCR can still accumulate at significant frequencies for 454 sequencing. Site-specific error may be a more useful comparison for different enzymes rather than mean substitution error, we therefore used a cut-off at which variants can reliably be called “real” as the upper 95% percentile of the error distribution (Table 4).

Library-based pyrosequencing involves shearing of PCR fragments prior to sequencing, and is faster than amplicons sequencing because it does not rely on multiple second-round primer sets. However, lower PCR efficiency is achieved with the larger amplicons used for library-based sequencing, and shearing often results in regions of low coverage, making it difficult to consistently detect low-level variants [33]. Another study utilizing library sequencing of HIV has noted uneven read coverage [2]. The latter paper showed large disparities in read numbers between different regions as well as between samples. We were able to improve coverage consistency for pyrosequencing by utilizing an overlapping amplicon approach, although the amplicon sizes chosen for our studies were too long, as they were based on expected but unrealized extensions in read lengths from recent advances in 454 sequencing chemistries.

An advantage of the amplicon approach is that it ensures that the universal adaptors A and B are properly incorporated onto each end of the amplicons, eliminating the need to select for DNA fragments with adaptors A and B in library preparation. It also permits the use of random bar codes onto template molecules to permit derivation of consensus sequences free of PCR and pyrosequencing errors when regions no longer than a single pyrosequencing read is being evaluated [23,24].

Although we addressed PCR mismatch error, we did not examine the role of reverse transcription of RNA into cDNA. Reverse transcriptases have higher inherent error than DNA polymerases [34], and a mismatch at that stage will be scored as real variation because it occurs in the template introduced into the PCR.

The impact of minor variants on disease progression and HIV evolution is controversial. In drug resistance studies, some have reported an association between minor variants and poor clinical outcome [35-40], while others have found no such association [41-44]. Another important application of MPS is for the detection of minor variants in acute HIV infection, and possibility of early evolution resulting from immune selection pressure as detectable viremia emerges [27]. Studies utilizing deep sequencing to study immune evasion have detected escape variants not found by Sanger sequencing that occur earlier and with greater complexity [1,2,45-47]. More work is needed to understand the significance of minor variants in HIV-1 evolution and clinical progression, and the protocols described herein will enhance our ability to probe diverse sequence populations more deeply.

## Supporting Information

Table S1 **Sample characteristics and sequencing of viral genome segments from 7 subjects.**
(DOCX)
