# Supplementary material for: Improved Detection of Rare HIV-1 Variants using 454 Pyrosequencing
Source: PLoS One. 2013 Oct 2;8(10):e76502. doi: 10.1371/journal.pone.0076502 (PMC3788733; doi:10.1371/journal.pone.0076502)
Supplement: Table S1 — Sample characteristics and sequencing of viral genome segments from 7 subjects. (DOCX) [file pone.0076502.s001.docx]

**Supplementary Table 1. Sample characteristics and sequencing of viral genome segments from 7 subjects**

**A. Total # of reads^1^**

**Plasma Mean amplifiable Amplicon**

**Sample ID viral load^2^ VL (range) p17 p24a p24b pol1 pol2 pol3 env1 env2 env3 env4 env5**

37628 11,934 7821 (4913-10730) 6844 15143 15584 23837 8994 11415 21650 78797 28969 49502 9526

11473 102,720 55821 (28082-83560) 25977 31265 22463 26331 34182 24052 25921 64439 33532 49175 19456

64236 11,330 1313 (955-1671) 7714 7880 8016 8538 8225 7057 8523 9280 10292 17765 8853

99203 2,308 312 (196-428) 6176 8478 5481 8331 9347 7546 11952 10834 11614 17255 15650

44149 4,100 1337 (1040-1634) 8949 5666 6451 7433 6510 11561 8131 15657 8527 12615 6528

68008 7,503 1306 (823-1789) 6716 5173 4619 8064 7547 5773 9309 9108 7801 15992 5043

1 Includes forward and reverse reads.

2 Number of viral RNA copies per ml of blood plasma determined using the Taqman assay.
